# Design of dual-diameter nanoholes for efficient solar-light harvesting

**DOI:** 10.1186/1556-276X-9-481

**Published:** 2014-09-11

**Authors:** Cheng Zhang, Xiaofeng Li, Aixue Shang, Shaolong Wu, Yaohui Zhan, Zhenhai Yang

**Affiliations:** 1 College of Physics, Optoelectronics and Energy & Collaborative Innovation Center of Suzhou Nano Science and Technology, Soochow University, Suzhou 215006, China; 2 Key Lab of Advanced Optical Manufacturing Technologies of Jiangsu Province and Key Lab of Modern Optical Technologies of Education Ministry of China, Soochow University, Suzhou 215006, China

**Keywords:** Photovoltaic device, Nanohole system, Light trapping, Light-conversion efficiency

## Abstract

A dual-diameter nanohole (DNH) photovoltaic system is proposed, where a top (bottom) layer with large (small) nanoholes is used to improve the absorption for the short-wavelength (long-wavelength) solar incidence, leading to a broadband light absorption enhancement. Through three-dimensional finite-element simulation, the core device parameters, including the lattice constant, nanohole diameters, and nanohole depths, are engineered in order to realize the best light-matter coupling between nanostructured silicon and solar spectrum. The designed bare DNH system exhibits an outstanding absorption capability with a photocurrent density (under perfect internal quantum process) predicted to be 27.93 mA/cm^2^, which is 17.39%, 26.17%, and over 100% higher than the best single-nanohole (SNH) system, SNH system with an identical Si volume, and equivalent planar configuration, respectively. Considering the fabrication feasibility, a modified DNH system with an anti-reflection coating and back silver reflector is examined by simulating both optical absorption and carrier transport in a coupled way in frequency and three-dimensional spatial domains, achieving a light-conversion efficiency of 13.72%.

**PACS:**

85.60.-q; Optoelectronic device; 84.60.Jt; Photovoltaic conversion

## Background

Efficient light trapping plays a significant role in realizing high-performance photovoltaic devices [1-8], including nanophotonic crystals, metallic (plasmonic) nanostructures, and nanotextured surfaces. In recent years, directly nanopatterning the photoactive materials into arrays of nanowires (NWs) [9,10], nanoholes (NHs) [11,12], nanocones (NCs) [13], or nano-cone-holes (NCHs) [14] has also attracted intensive attention. Through unveiling the controllability of diameter and depth of NWs on the optical absorption, Hu and Chen proposed that NWs are good candidates for photovoltaics [9]. Lin and Povinelli demonstrated that NWs with a large lattice constant have an outstanding optical absorption arising from the increased field concentration and the excitation of guided resonant modes [10]. NHs were later investigated by Han and Chen as an alternative light absorber that was proved to be superior to nanorod arrays [11].

Recently, Hua et al. investigated the absorption of multi-diameter nanopillars (MNPLs) systematically and showed that the absorption approaches that of NCs by increasing the number of nanopillar layers [15]. Furthermore, Fan et al. showed that dual-diameter NWs with a small-diameter tip and a large-diameter base exhibit an enhanced absorption over the wavelength range of 300 to 900 nm [16]. As NHs show superiority over NWs in light trapping, here, we study the possibility of further performance optimization of solar cells with NHs.

In this paper, through carefully examining the absorbing capability of NHs with different diameters, we propose the photovoltaic system composed of dual-diameter NHs (DNHs) with a large diameter at the top and a small diameter at the bottom, enabling broadband and strong optical absorption. The lattice constant, diameters, and depth ratio of the top and bottom NHs have been extensively studied in order to maximize the light absorption of the new system. The underlying physics have been explained by examining the absorption spectra as well as the spatial characteristics of the optical absorption. Our study indicates that the new stand-alone DNH system exhibits improved performance than previous single-diameter NHs (SNHs), NWs, and planar setups. Finally, the DNH design with the front anti-reflection coating and a back silver reflector is studied. Optoelectronic simulation allows us to evaluate the optical and electrical performance in a comprehensive way. It is predicted that the light-conversion efficiency can be up to 13.72%.

## Methods

**Figure 1:**
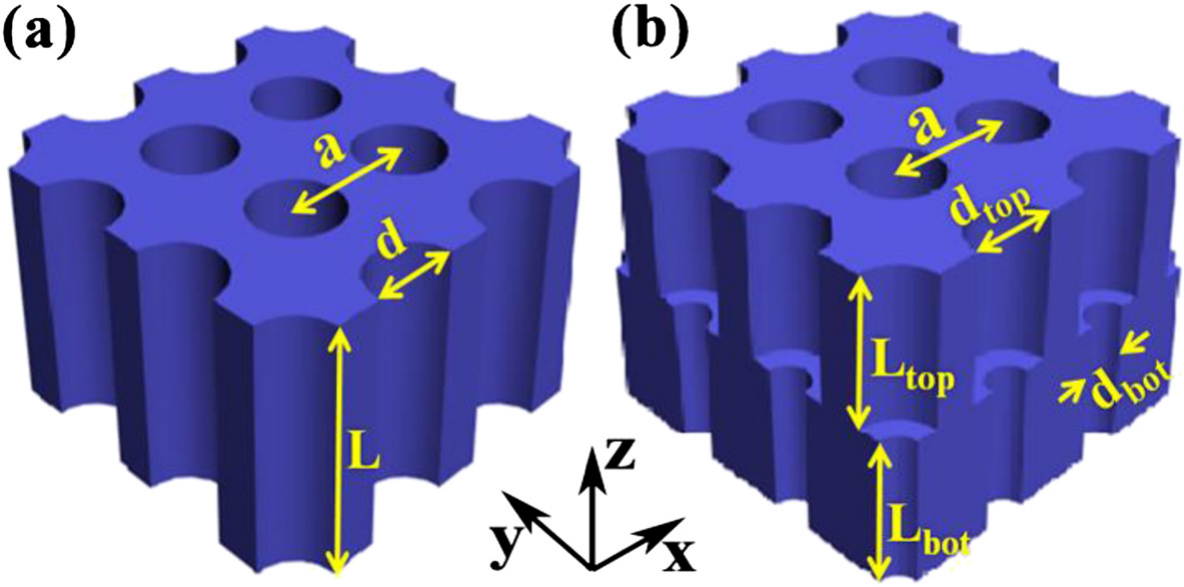
**Device. (a)** SNHs and **(b)** DNHs, where the geometrical parameters have been shown.

Figure 1 shows the schematic of the nanostructured photovoltaic system composed of (a) SNHs and (b) DNHs arranged in a square lattice (lattice constant *a*) with holes filled by air. The sunlight is incident on the system normally. The geometrical parameters have been inserted into the figure. The total hole depth (*L* for SNHs; *L*_top_ + *L*_bot_ for DNHs) is fixed at 2.33 μm for a fair comparison with those of [9,11]. Based on the optical constants from Palik [17], simulations are performed by finite element method [18] with a maximum mesh size of 60, 28, and 40 nm for air, top silicon, and bottom silicon, respectively. With the calculated absorption spectrum, the maximum short circuit-current density under a perfect internal quantum process [11] is obtained by the equation: $J_{\mathrm{SC}}=\frac{e}{\mathrm{hc}}\int_{310\;\mathrm{nm}}^{\lambda_{g}}I\left(\lambda \right)A\left(\lambda \right)\lambda d\lambda$. The ultimate efficiency *η* which quantifies the overall absorption performance is given then by $\eta =\frac{\int_{310\;\mathrm{nm}}^{\lambda_{g}}I\left(\lambda \right)A\left(\lambda \right)\frac{\lambda }{\lambda g}d\lambda }{\int_{310\;\mathrm{nm}}^{4,000\;\mathrm{nm}}I\left(\lambda \right)d\lambda }$, where *e* is the electron charge, *h* is the Planck constant, *c* is the light velocity in vacuum, *λ*_g_ = 1,127 nm is the wavelength corresponding to the bandgap energy of silicon, *I*(*λ*) is the AM 1.5G standard solar irradiation spectrum [19], and *A*(*λ*) is the absorptance. The spectral range is from 310 to 1,127 nm to characterize the light-matter coupling between silicon and solar incidence.

Keeping the fabrication feasibility in mind, an 80-nm ITO layer (working as the anti-reflection layer and contact) and a back silver reflector are introduced. The final photocurrent response of the new DNH solar cells through a detailed investigation on the internal carrier transport and recombination process is performed (see our previous publications [5,20-22]). The optoelectronic simulations include carrier generation, recombination, transport, and collection mechanisms with the carrier generation profile taken from the electromagnetic calculation. In this way, the actual external quantum efficiencies (EQEs) and the short-circuit photocurrent densities of the conventional SNH and the new DNH devices can be obtained. With the dark current response calculated [21] as well as the series and shunt resistances estimated from the experiment, the current-voltage (*J*-*V*) curve is achieved, allowing to evaluate the cell performances, such as open-circuit voltage (*V*_oc_) and actual light-conversion efficiency.

## Results and discussion

**Figure 2:**
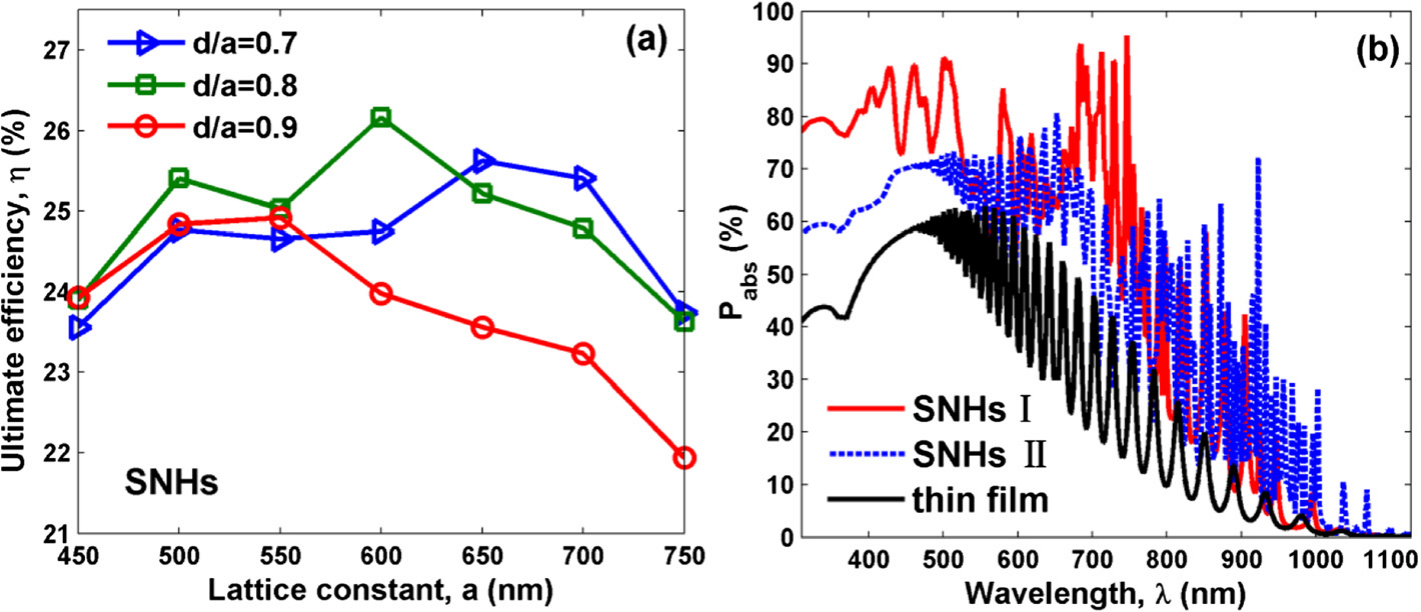
**SNHs lattice constant, opening ratio optimization, and absorption spectra. (a)** The ultimate efficiency of SNHs as a function of lattice constant *a* and hole opening ratio *d*/*a*. **(b)** Absorption spectra of SNHs I (*a* = 600 nm, *d*/*a* = 1) and SNHs II (*a* = 600 nm, *d*/*a* = 0.5). The thin film absorption spectrum is inserted as a reference.

Figure 2a shows the dependence of the ultimate efficiency *η* on the lattice constant (*a*) and hole opening ratio (*d/a*). According to [14], the optimal lattice constant and filling ratio (*f)* of SNHs are about 600 nm and 0.5 (corresponding to *d*/*a* = 0.8), respectively, so *d/a* is limited from 0.7 to 0.9. For each *d/a*, *η* tends to rise and then drop with increasing *a*. The optimization occurs at *a* = 600 nm and *d* = 480 nm, yielding *η* = 26.17%, which is close to that reported in [14] (i.e., 26.52% when *a* = 600 nm and *f* = 0.5, i.e., *d/a* = 0.8).

Although with a high efficiency by SNHs, it is believed that the light-conversion capability of nanoholed photovoltaic system can be further improved with a proper modification of the device configuration. We therefore examine the absorption spectra of SNHs under two configurations, i.e., 1) large holes (*d*/*a* = 1) and 2) small holes (*d*/*a* = 0.5), as depicted in Figure 2b where the planar case (thickness = 2.33 μm) is also attached for comparison. It is observed that the absorption resonances exhibit obvious red shifts as *d*/*a* reduces [14]. This implies that SNHs with large holes show higher (weaker) light absorption in the short-wavelength (long-wavelength) region, while SNHs with smaller holes behave in a contrary way. The special diameter dependency can be interpreted by the fact that the effective refractive index of SNH system with large holes is relatively low (better impedance-matched with air), enabling more light entering the cell and greatly strengthening the light absorption in short-wavelength band [13]; nevertheless, the reduced active silicon material could not thoroughly absorb the long-wavelength light due to the much degraded material extinction coefficient [17].

The special relation between the spectral absorption behavior and physical size of NHs provides an effective way of managing and realizing a broadband optical absorption. Normally, short-wavelength (long-wavelength) light is absorbed by the region close to the top (bottom) facet. Therefore, we propose here a dual-layer nanoholed system (DNHs) which is composed by a top NH layer with large holes and a bottom NH layer with small holes. The dual-layer setup takes advantage of the super capability of NH systems with different hole sizes in absorbing solar incidence at specified spectral bands and thus contributes to a broadband enhancement.

**Figure 3:**
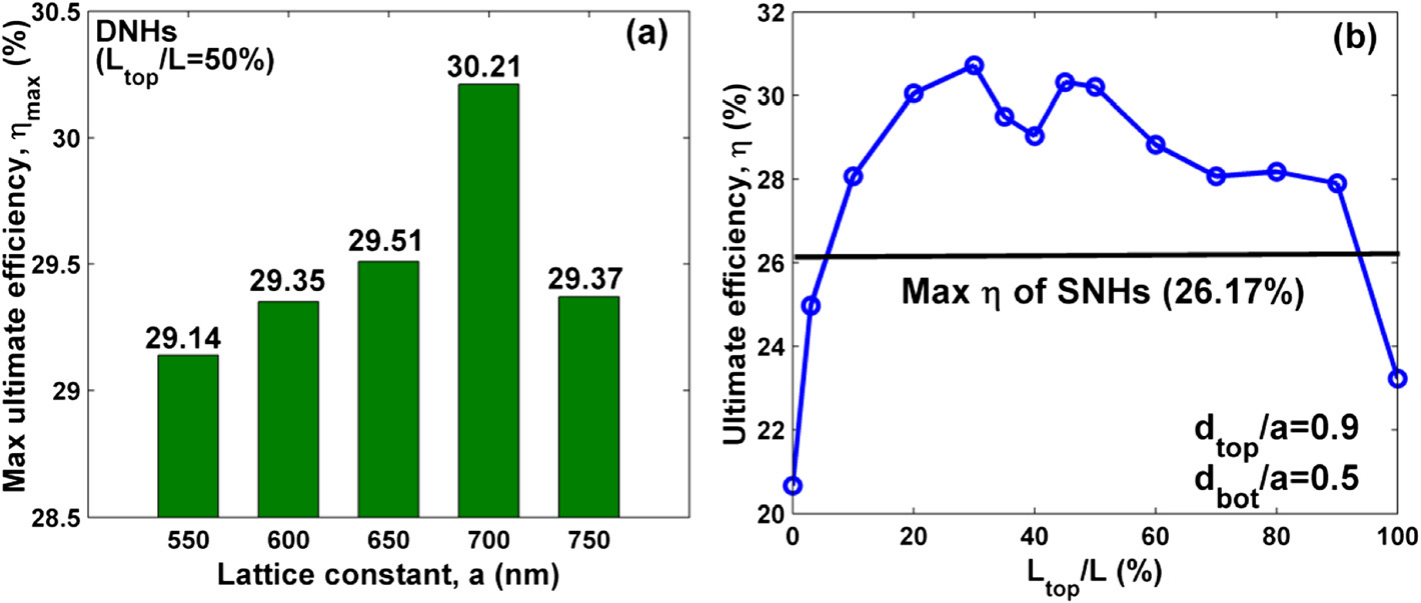
**DNHs optimization. (a)** Maximized ultimate efficiency of the DNHs as a function of lattice constant *a* with *L*_top_/*L* = 50%. **(b)** Ultimate efficiency versus *L*_top_/*L* at *a* = 700 nm, *d*_top_/*a* = 0.9, and *d*_bot_/*a* = 0.5.

We first examine the possibility of achieving a higher *η* from the dual-diameter NH photovoltaic system by properly engineering the lattice constants and hole diameters of both the top and bottom NH layers. In this study, it is assumed that two NH layers have an identical lattice constant, but the diameters (*d*_top_ and *d*_bot_) can be artificially controlled in order to maximize *η*. The results are listed in Figure 3a, where the highest *η* has been recorded for each *a* after a large number of three-dimensional optimizations by sweeping the top and bottom hole diameters (*d*_top_/*a* from 0.7 to 1 and *d*_bot_/*a* from 0.2 to 0.7). In addition, the hole depth ratio is temporarily fixed to be *L*_top_/*L* = 0.5. It is found that the *η* of DNHs can be up to 30.21% when *a* = 700 nm, *d*_top_/*a* = 0.9, and *d*_bot_/*a* = 0.5. It is much higher than the best value of SNHs (26.17%) [14]. In fact, although with a high efficiency, the DNH system still leaves a room for further improvement by optimally setting the thickness ratio between the top and bottom NH layers. Figure 3b shows the tunability of *η* by adjusting *L*_top_/*L*, which is considered to be from 0% (with only bottom NHs) to 100% (with only top NHs). The other parameters are borrowed from the design of Figure 3a. As displayed, the DNH system is usually better than SNH and shows the further increased *η* = 30.72% (i.e., *J*_sc_ = 27.93 mA/cm^2^) when *L*_top_/*L* = 30%, *a* = 700 nm, *d*_top_/*a* = 0.9, and *d*_bot_/*a* = 0.5. Actually, compared to the best SNHs (see the black line inserted in Figure 3b), there is a very wide range for *L*_top_/*L* (i.e., 7% ~ 92%) to let the DNHs outperform the SNHs for solar applications.

**Figure 4:**
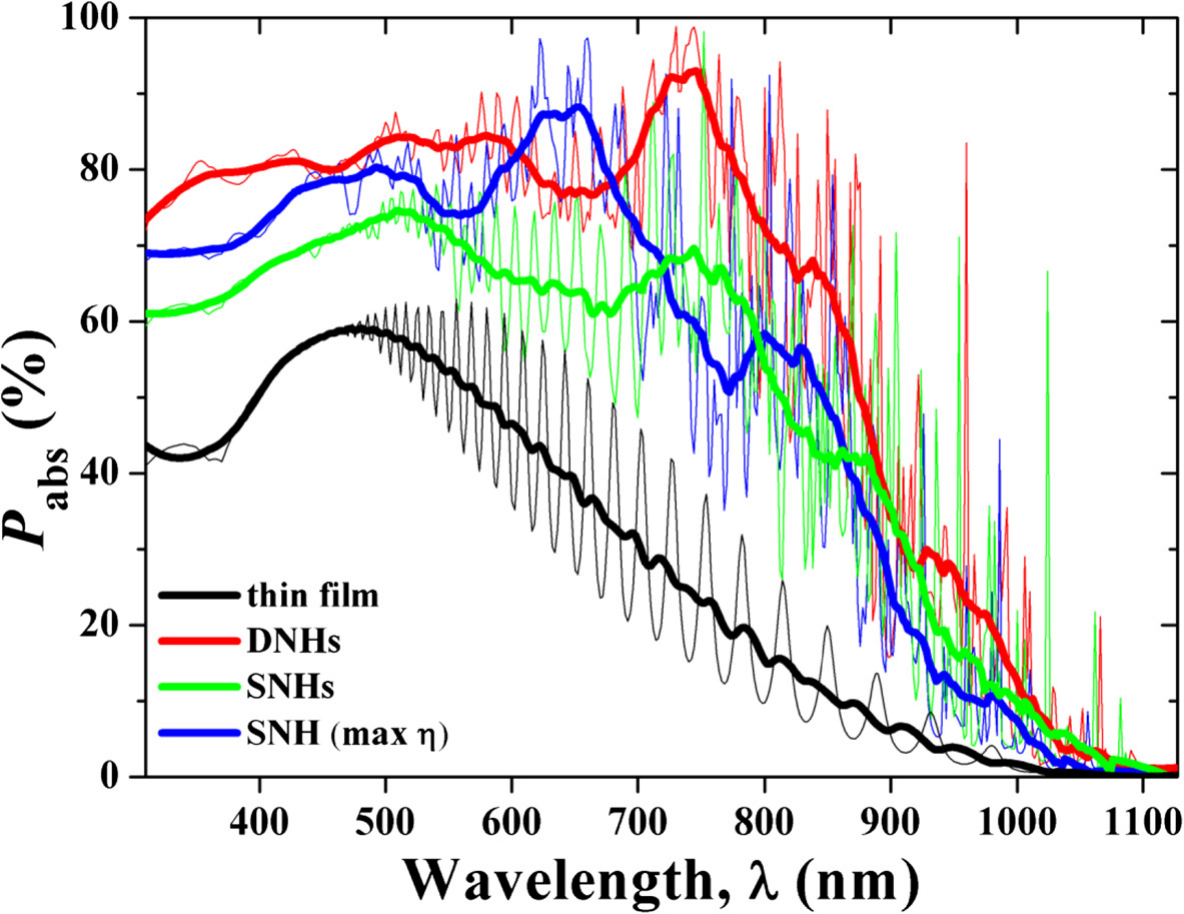
**Absorption spectra.** Absorption spectra of the optimized DNH (*L*_top_/*L* = 30%, *a* = 700 nm, *d*_top_/*a* = 0.9, and *d*_bot_/*a* = 0.5), SNH with an identical Si volume, and thin film systems with an identical Si thickness. The best SNH system with *a* = 600 nm and *d* = 480 nm is also inserted for comparison. The thin lines are the calculated absorption spectra, and the thick ones are the smoothed versions.

Figure 4 compares the spectral responses (absorption percentage (*P*_abs_)) of the photovoltaic systems under various nanoconfigurations (see figure caption for details). Although nanostructured photovoltaic systems show much improved ability of harvesting the solar incidence in the whole spectrum [11,12], DNHs exhibit the highest and most broadband absorption. Under the combined merits of large and small NHs, an ultra-broadband absorption enhancement is achieved except for a narrow band centered at around 650 nm, where the strong cavity resonances excited from the SNH system. The higher and broadband absorption enhancement results from the cavity resonant peaks excited in the DNH design. For example, a distinct peak *P*_abs_ over 80% can be seen at the wavelength around 960 nm near *λ*_g_, which is significantly higher than other cases, e.g., *P*_abs_ = 27.77% for the best SNHs. These results validate our initial consideration on the design of dual-diameter NHs and unveil the physics behind the absorption enhancement mechanisms. It is noted that if we compare the ultimate efficiency of the novel DNH system with the planar configuration under an identical Si thickness, the enhancement ratio of *η* is over 100%, as easily predicted from Figure 4.

**Figure 5:**
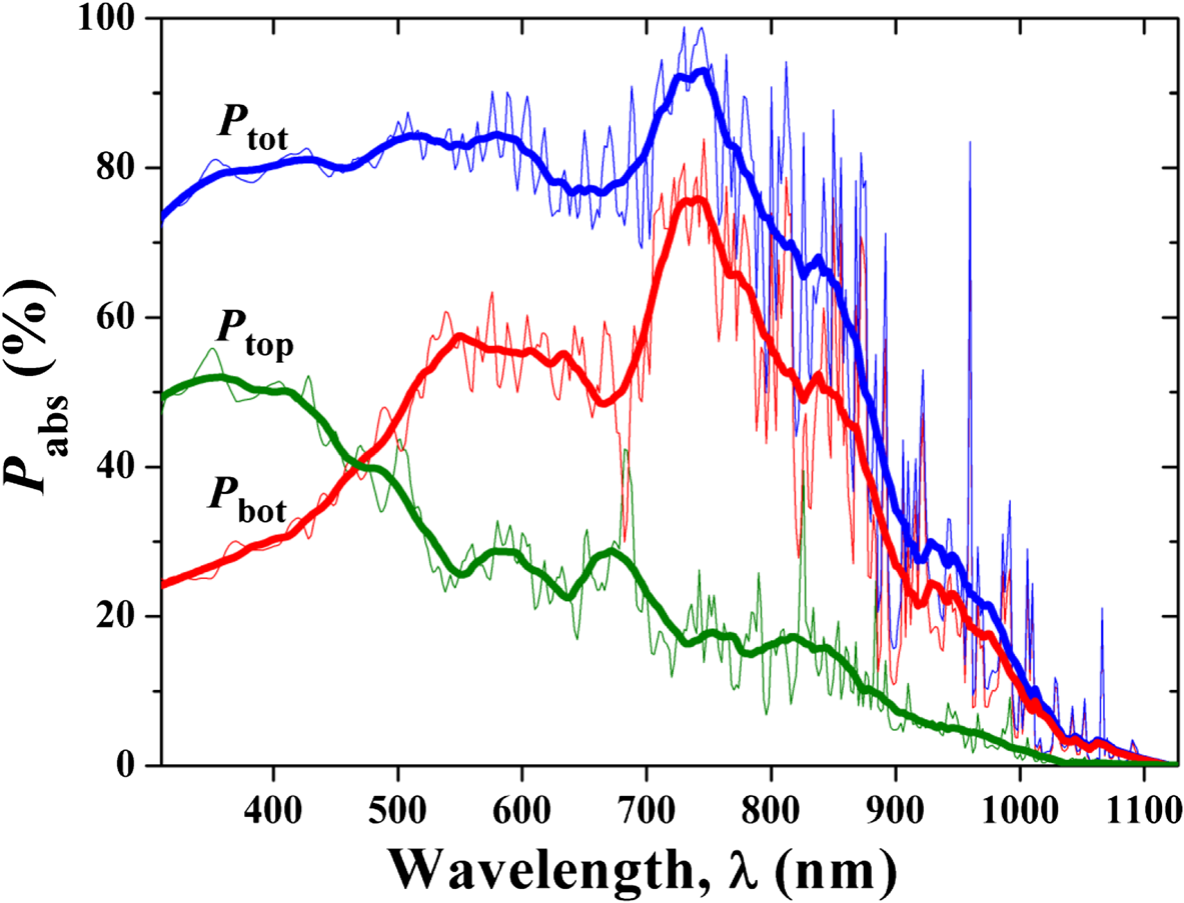
**Absorption spectra.** Absorption characteristics of the top/bottom nanoholes in the optimized DNHs. The absorption percentages of the total system, top NH layer, and bottom NH layer are defined here as *P*_tot_, *P*_top_, and *P*_bot_, respectively. The thin lines are the calculated absorption spectra, and the thick ones are the smoothed versions.

**Figure 6:**
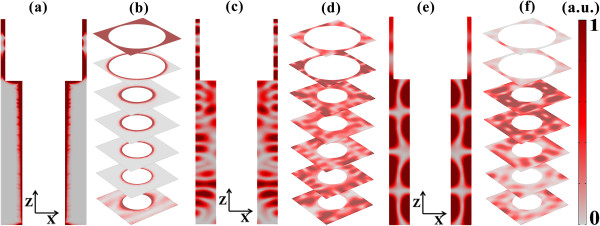
**Optical absorption distributions.** Spatial profiles of optical absorption in the optimized DNHs at *λ* = 350 nm **(a, b)**, 700 nm **(c, d)**, and 960 nm **(e, f)**.

It is now necessary to distinguish the absorption contributions from the top and bottom NH layers in order to thoroughly understand the optical response (see Figure 5). It is shown that the majority of the incidence is absorbed by the bottom NH layer; however, the top NH layer dominates the absorption when *λ* < 460 nm as expected. Comparing the absorption spectra of the top and bottom NHs in long-wavelength region (*λ* > 800 nm), the bottom NH layer absorbs more light than the top, showing that the total DNH absorption peaks are actually mainly contributed from the bottom NH layer with small holes (in other words, larger Si volume). For an optical insight, the absorption coefficient of silicon to the long-wavelength light is very low, allowing the light to propagate into and being absorbed by the bottom NH layer. Moreover, the volume of the bottom small-NH layer is larger than that of the top large-NH layer, which further improves the absorption of the bottom layer for the long-wavelength light. The absorption patterns at typical wavelengths (350, 700, and 960 nm) are shown in Figure 6. These patterns well illustrate the localized absorption inside the NH cavity and satisfactorily verify the previous observation and explanation. For example, under short-wavelength incidence, the light is mainly absorbed by the top NH layer and the region close to the hole (Figure 6a,b); under long-wavelength incidence, light penetrates into the Si volume and forms strong cavity resonant peaks. A distinct cavity effect can be seen from Figure 6e,f, where the strong Fabry-Perot resonant pattern is observed inside the bottom NH cavity, leading to the prominent peak around 960 nm as shown in Figure 4.

**Figure 7:**
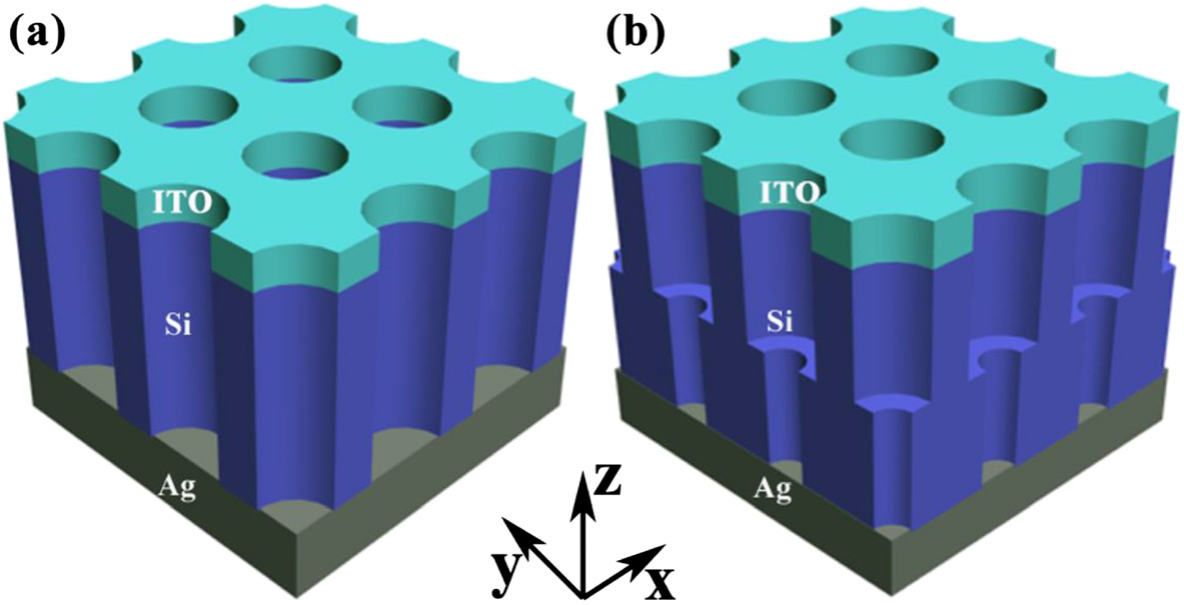
**Realistic photovoltaic systems.** SNH **(a)** and DNH **(b)** photovoltaic systems with an 80-nm front anti-reflection layer and a rear metallic reflector. The substrate is not shown in this figure.

Finally, considering the realistic photovoltaic configurations, an ITO layer with a thickness of 80 nm, and a thin silver coating on the rear side are introduced into the optimal SNH and DNH systems (see Figure 7), to function as the contact, anti-reflection layer, and the back reflector, respectively. To evaluate the electrical response of the realistic devices, detailed simulations considering both optical absorption and carrier transport are performed [5,21]. The axial semiconductor junction is configured as follows: the donor (acceptor) concentration is 1.6 × 10^20^ (1 × 10^18^) cm^-3^, the n- and p-type regions are with an identical thickness of 150 nm, and the rest belongs to the intrinsic region. More parameters can be obtained from [23].

**Figure 8:**
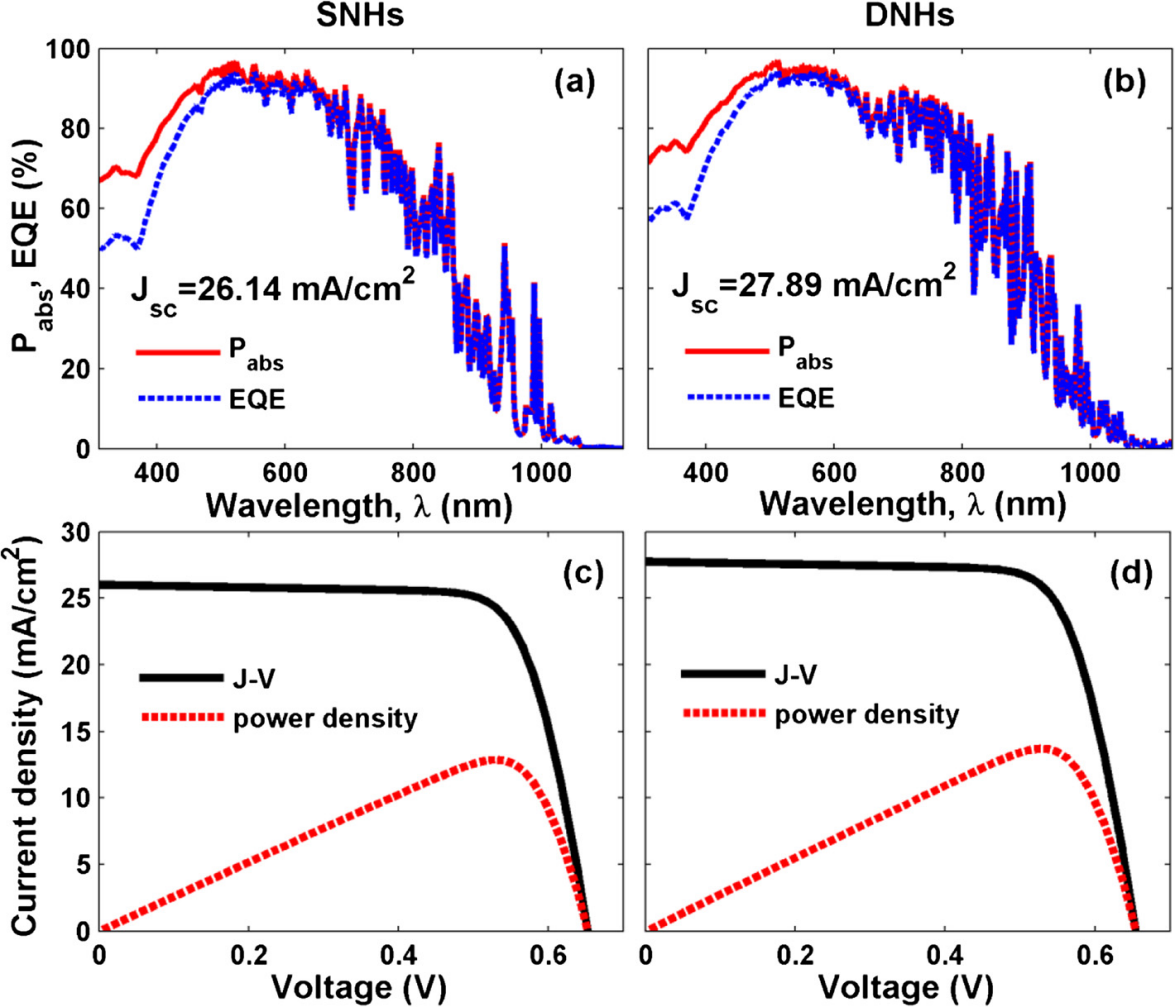
**EQE spectra and *J*-*V *characteristics.** The best SNH **(a)** and optimized DNH **(b)** photovoltaic systems with an 80-nm front anti-reflection layer and a rear metallic reflector. Corresponding *J*-*V* characteristics **(c, d)**.

Figure 8 shows the *P*_abs_ and EQE spectra of the realistic SNH (a) and the newly designed DNH (b) systems. Compared to *P*_abs_ in Figure 4, the absorptance in the short-wavelength range increased obviously due to the anti-reflection effect [3]. In addition, the light absorption in the long-wavelength range also increased on account of the re-absorption of the unabsorbed solar incidence in the first light path, which has been reflected back into the active region by the rear Ag reflector [1], although several narrow and strong absorption peaks in Figure 4 have disappeared in Figure 8b as the system configuration has been changed. The actual imperfect internal quantum process caused by the surface recombination and other carrier loss mechanisms results in a great degradation on the electrical properties of the device, implied as a big discrepancy between *P*_abs_ and EQE especially in the short-wavelength range. Through the spectral integration to the EQE spectra, *J*_sc_ of the realistic SNH and DNH devices are 26.14 and 27.89 mA/cm^2^ (please note that the values directly converted from *P*_abs_, i.e., without electrical simulation, are 27.06 and 28.66 mA/cm^2^), respectively.

The corresponding *J*-*V* characteristics are plotted in Figure 8c,d, based on the previously calculated *J*_sc_ and the dark current densities under continuously increasing forward electric bias (*V*). The series and shunt resistances (*R*_s_ and *R*_sh_) can be roughly estimated by extracting the slope information from the *J*-*V* curve at the point of *V* = 0 and *V*_oc_[24]. The values of *R*_s_ and *R*_sh_ are estimated to be around 2 and 1,000 Ω cm^2^, respectively, by using the *J*-*V* curve from [25]. From the illustration, performance parameters like maximum output power density (*P*_max_), *V*_oc_, fill factor (FF = *P*_max_/(*J*_sc_*V*_oc_), and the actual light-conversion efficiency can be obtained. It is found that *J*_sc_ is increased without degrading *V*_oc_ (*V*_oc_ = 0.65 V), leading to a higher *P*_max_. Under a FF of approximately 76.01%, the light-conversion efficiency is predicted to be 13.72%. The actual efficiency is much less than the ‘ultimate efficiency’ from pure optical prediction since the carrier recombination and resistance losses are included.

## Conclusions

We report the possibility of dramatically enhanced absorption of solar irradiance by using dual-diameter nanohole photovoltaic arrays. Broadband absorption enhancement is achieved by combining the merits of both small and large nanoholes which are spatially separated in different layers. Through a systematical optimization on the lattice constant, nanohole diameters, and depths of top and bottom NHs, the DNH photovoltaic system is designed to show a much improved ultimate efficiency up to 30.72% (i.e., *J*_sc_ = 27.93 mA/cm^2^), which is 17.39% (26.17%) higher than the best SNHs (SNHs with an identical Si volume). The novel DNH system shows light-absorbing capability which has doubled that based on conventional planar configuration. The better impedance match in the front surface and strengthened resonant cavity modes are responsible for the broadband enhancement in various wavelength regions. For a conventional realistic solar cell design, an 80-nm ITO layer and an Ag back reflector are included into the DNH system. Considering both optical and electrical perspectives, the realistic DNH device performs 13.72% light-conversion efficiency.

## Abbreviations

DNH: dual-diameter nanohole; EQEs: external quantum efficiencies; FF: fill factor; *J*-*V*: current-voltage; MNPLs: multi-diameter nanopillars; NCs: nanocones; NCHs: nano-cone-holes; NHs: nanoholes; NWs: nanowires; *P*_max_: maximum output power density; *R*_s_: series resistances; *R*_sh_: shunt resistances; SNH: single nanohole; *V*_oc_: open-circuit voltage.

## Competing interests

The authors declare that they have no competing interests.

## Authors' contributions

CZ carried out the design and drafted the manuscript. XL conceived the design and supervised the research. AS and ZY participated in the *J*-*V* simulation. YZ and SW commented on the results and revised the manuscript. All authors read and approved the final manuscript.
